# *Impatiens leshanensis* (Balsaminaceae), a new species from Sichuan, China

**DOI:** 10.3897/phytokeys.274.190265

**Published:** 2026-05-04

**Authors:** Xingyu Du, Yanru Zhang, Shihao Jiang, Qinyi Wang, Jiazhen Wu, Bo Li, Yan Chen, Donglai Hua, Bing Zhang, Xianhua Gu, Ke Huang, Zhixi Fu

**Affiliations:** 1 Key Laboratory of Land Resources Evaluation and Monitoring in Southwest, Sichuan Normal University, Ministry of Education, Chengdu 610066, China School of Life Sciences (School of Ecological Forestry), Mianyang Normal University Mianyang China; 2 Sichuan Ecological Environment Monitoring Station, Chengdu 610091, China Key Laboratory of Land Resources Evaluation and Monitoring in Southwest, Sichuan Normal University Chengdu China; 3 Leshan Eco-environmental Monitoring Center Station of Sichuan Province, Leshan 614000, Sichuan, China College of Life Sciences, Sichuan Normal University Chengdu China; 4 School of Life Sciences (School of Ecological Forestry), Mianyang Normal University, Mianyang 621000, China Sichuan Ecological Environment Monitoring Station Chengdu China; 5 Chengdu Xing'ai Information Technology Co., Ltd., Chengdu 610051, China Leshan Eco-environmental Monitoring Center Station of Sichuan Province Leshan China; 6 College of Life Sciences, Sichuan Normal University, Chengdu 610101, China Chengdu Xing'ai Information Technology Co., Ltd. Chengdu China; 7 Sichuan Guoce Testing Technology Co., Ltd., Chengdu 610023, China Sichuan Guoce Testing Technology Co., Ltd. Chengdu China

**Keywords:** Balsaminaceae, morphology, new species, phylogeny, Sichuan

## Abstract

*Impatiens leshanensis* X.H.Gu, K.Huang & Z.X.Fu, **sp. nov**., a new species of Balsaminaceae from Jinkouhe District, Leshan City, Sichuan Province, China, is described and illustrated. Phylogenetic analysis based on the complete cp genome indicates that the new species is closely related to *Impatiens faberi* Hook.f., forming a monophyletic clade. However, *I. leshanensis* can be distinguished from *I. faberi* and other morphologically similar species (including *Impatiens baishaensis* B.Ding & H.P.Deng, *Impatiens piufanensis* Hook.f., *Impatiens forrestii* Hook.f. & W.W.Sm, *Impatiens distracta* Hook.f., *Impatiens lateristachys* Y.L.Chen & Y.Q.Lu, *Impatiens rostellata* Franch., *Impatiens rectirostrata* Y.L.Chen & Y.Q.Lu, and *Impatiens longcanggouensis* Q.Luo.) by its unique morphological traits: oblong-elliptic dorsal petal as wide as the lateral petals, narrowly funnelform lower sepal, pale pink flowers with deep red spots and pink stripes, and ellipsoid seeds with reticulate ornamentation. Additionally, the distribution map and chloroplast genome features are provided.

## Introduction

The genus *Impatiens* L. is a large genus in the family Balsaminaceae with over 1000 species worldwide (Hooker 1908; Chen 1978; Yu et al. 2016; Zhao et al. 2020). It is highly valued for ornamental purposes due to its showy flowers and unique floral morphologies, and is often referred to as "the orchids of dicotyledons" (Yuan et al. 2004; Yu et al. 2016). The genus *Impatiens* L. is mainly distributed in tropical and subtropical mountainous regions of the Old World, with their diversity particularly concentrated in five key areas: Tropical Africa, Madagascar, South India-Sri Lanka, eastern Himalayas, and Southeast Asia (Song et al. 2003; Yuan et al. 2004).

In addition to *Impatiens*, recent years have witnessed the discovery of numerous new species across other angiosperm families in China (Hu et al. 2017; Chen et al. 2017a, 2017b; Qiu et al. 2024a; Yang et al. 2024). China is one of the important modern distribution and diversification centers of *Impatiens*, with over 350 recorded wild species, most of which are concentrated in southwestern provinces and autonomous regions such as Yunnan, Sichuan, Tibet, and Guizhou (Yuan et al. 2022b), exhibiting extremely high species endemism (Yu 2012). As an important distribution center of *Impatiens* in China, Sichuan Province harbors rich and diverse wild *Impatiens* species, totaling about 108 taxa (including 1 variety). Among these, 94 are Chinese endemic species and 41 are Sichuan endemic species. These species are primarily distributed in the transitional zone from the western Sichuan Plateau to the southwestern mountains (Cen et al. 2025). However, traits of *Impatiens* impede specimen preservation and species identification (including succulent stems and leaves, fragile flowers, and explosive capsules). They bring great challenges to taxonomic research (Chen 1978; Yu 2012; Narayanan et al. 2013).

In recent years, numerous new *Impatiens* species have been reported in China. These include *Impatiens bijieensis* X.X.Bai & L.Y.Ren (Ren et al. 2022), *Impatiens liupanshuiensis* X.X.Bai & T.H.Yuan (Yuan et al. 2022a), *Impatiens nushanensis* Z.Wang, P.P.Wu & S.X.Yu (Wang et al. 2022a), *Impatiens chenmoui* Zheng W.Wang, X.C.Li & Q.Wang (Wang et al. 2022b), *Impatiens longyangensis* Y.Y.Cong, G.W.Hu & S.Peng (Hu et al. 2022), *Impatiens yaojiapingensis* Y.Y.Cong, G.W.Hu & T.Hu (Hu et al. 2022), *Impatiens yunlingensis* S.X.Yu, Chang Y.Xia & J.H.Yu (Yu et al. 2022), *Impatiens cavaleriei* X.X.Bai & R.X.Huang (Huang et al. 2023), *Impatiens spiralis* Y.Y.Cong, G.L.Zhang & Y.M.Zheng (Zheng et al. 2023), *Impatiens mogangensis* Y.M.Shui & W.H.Chen (Lyu et al. 2024), *Impatiens beipanjiangensis* Jian Xu & H.F.Hu (Hu et al. 2024), *Impatiens lhunzeensis* J.Tian, G.W.Hu & Q.F.Wang (Tian et al. 2024), *Impatiens uncata* Y.Y.Cong & J.J.Zhou (Qiu et al. 2024b), *Impatiens fujianensis* Liang Ma, Xin Y.Chen & S.P.Chen (Ma et al. 2024), *Impatiens maolanensis* Z.B.Xiong & Q.Y.Wen (Li et al. 2025), *Impatiens glandulosocalycina* Y.Y.Cong, G.W.Hu & T.Hu (He et al. 2025), and *Impatiens tainingensis* J.D.Lin & P.Li (Wang et al. 2026). Among them, numerous new *Impatiens* species have also been reported in Sichuan Province. These include *Impatiens sikaiensis* Q.Luo & Y.Yuan (Ying et al. 2023), *Impatiens zhaojueensis* Q.Luo (Qiang et al. 2023), *Impatiens yingjingensis* X.Q.Song, B.N.Song & Biao Yang (Song et al. 2024), *Impatiens yixiangensis* Han W.Huang & R.Li (Huang et al. 2025a), *Impatiens meishanensis* K.Huang & Z.X.Fu (Zhang et al. 2025), *Impatiens amphitricha* H.W.Huang & R.Li (Huang et al. 2025b), and *I. longcanggouensis* (Luo et al. 2026). Most of these newly described species have been recognized by both genetic data and morphological evidence.

In early October 2025, a distinctive *Impatiens* species was collected during field monitoring in the Bayuelin Nature Reserve of Leshan City, Sichuan Province. This species is morphologically similar to *I. baishaensis*, but can be distinguished as a new species by a suite of unique characters: oblong-elliptic dorsal petal as wide as the lateral petals, a narrowly funnelform lower sepal, pale pink flowers with deep red spots and pink stripes, and ellipsoid seeds with reticulate ornamentation. However, following detailed morphological comparison and phylogenetic analysis based on chloroplast genome, it did not match any previously described species, supporting its status as a new species within the genus *Impatiens*. Accordingly, we describe and illustrate the new species here.

## Materials and methods

### Morphological analysis

This study combined field observations with specimen comparisons. Specimens were collected in October 2025 from Jinkouhe District, Leshan City, Sichuan Province, China. Following the meticulous on-site dissection of the new species specimens, a thorough inspection of their morphological traits was performed. The investigation focused on petiole length, leaf length and width, the number of lateral vein pairs, adaxial and abaxial leaf surface colors, overall leaf morphology, floral morphology, seed shape and size under natural growth conditions.

Specimens were compared with those available in online herbaria, including the Chinese National Herbarium (**PE**) (https://pe.ibcas.ac.cn/index.html), JSTOR Global Plants (http://plants.jstor.org/), China Virtual Herbarium (**CVH**) (https://www.cvh.ac.cn/index.php), Royal Botanical Garden Edinburgh (**E**) (https://data.rbge.org.uk/search/herbarium/), and Kunming Institute of Botany, Chinese Academy of Sciences (**KUN**) (http://www.ui92.com/demo/html/1621/). Furthermore, following the preliminary identification of the new species, detailed observations and comparisons were performed with congeneric species, such as *I. faberi*, *I. baishaensis*, *I. piufanensis*, *I. forrestii*, *I. distracta*, *I. lateristachys*, *I. rostellata*, *I. rectirostrata*, and *I. longcanggouensis*.

Holotype specimens are deposited at the Herbarium of Sichuan Normal University (herbarium code: SCNU!). The conservation status assessment of the new species follows the criteria of the IUCN Red List of Threatened Species (IUCN 2024). The terminology system proposed by Yu et al. (2016) was adopted for the description of floral organs and discussions on ontogeny.

### DNA extraction and sequencing

Total genomic DNA was obtained through the utilization of the CTAB method (Doyle and Doyle 1987). Paired-end DNA libraries were constructed following the Illumina DNA Library Preparation Guide (Allen et al. 2006). The complete chloroplast genome was sequenced on the Illumina HiSeq XTen sequencing platform (San Diego, CA, USA). Genome assembly was performed using GetOrganelle v.1.7.7.1 (Jin et al. 2020; Qian et al. 2024), a method previously successfully applied in recent studies on the genus *Nymphaea* (Wang et al. 2022b; Song et al. 2024). The genome assembly results were verified using Bandage v.0.8.1 (Wick et al. 2015; Zhao et al. 2020).

Genome annotation was performed using CPGAVAS2 (Shi et al. 2019; Yan et al. 2023), subsequently complemented with manual verification and correction. The fully annotated genome was deposited in NCBI GenBank, with the accession number PX764269. Additionally, Chloroplot (https://irscope.shinyapps.io/Chloroplot) was employed to construct a visualization map of the chloroplast genome structure (Zhao et al. 2020), while CPGView (http://47.96.249.172:16085/cpgview/view) was utilized to characterize the relevant information of cis-splicing and trans-splicing (Li et al. 2024).

### Phylogenetic analysis

Altogether, 45 chloroplast genomes of *Impatiens* and two outgroups from Primulaceae and Ebenaceae were obtained from the NCBI GenBank database (https://www.ncbi.nlm.nih.gov/Genbank), with the detailed information of sequence sources summarized in Table 1. *Diospyros hainanensis* (Ebenaceae) and *Primula vialii* (Primulaceae) were selected as outgroups based on broader Ericales phylogenies, which place Ebenaceae and Primulaceae as closely related families to Balsaminaceae within the order Ericales. MAFFT v7.526 (Katoh and Standley 2013; Wu et al. 2021) was employed to perform multiple sequence alignment.

**Table 1.** Comparison of morphological characters among *Impatiens leshanensis*, *I. faberi*, *I. baishaensis*, *I. piufanensis*, *I. forrestii*, *I. distracta*, *I. lateristachys*, *I. rostellata*, *I. rectirostrata*, and *I. longcanggouensis*.

Characters	*I. leshanensis*	*I. faberi*	*I. baishaensis*	*I. piufanensis*	*I. forrestii*	*I. distracta*	*I. lateristachys*	*I. rostellata*	*I. rectirostrata*	*I. longcanggouensis*
Plant height (cm)	40–70	60–70	30–70	20–40	35–90	30–60	40–100	40–60	30–50	30–60
Shape of leaves	narrowly ovate or lanceolate	ovate-lanceolate or elliptic	ovate-oblong	Long, ovate, or lanceolate	ovate-lanceolate or subelliptic	ovate-oblong	broadly lanceolate	ovate or elliptic	ovate	lanceolate or ovate-lanceolate
Leave size (cm)	3–8 × 1–3	5–15 × 2.5–4.5	3–8 × 1–3.5	3–6 × 1.2–2.5	10–15 × 3–5	5–10	5–15 × ca. 6	3–4 × 2–2.5	10–14 × ca. 5	4–16 × 1–4
Leave margin	crenate or crenate-serrate	serrate or crenate-serrate	crenate	serrate	regularly serrate	crenate teeth or crenate serrations	serrate	crenate-serrulate	serrate	shallowly arcuate-serrate
Lateral veins	5–7 pairs	5–8 pairs	No data	4–5 pairs	8–9 pairs	6–7 pairs	6–8 pairs	4–5 pairs	5–7 pairs	6–9 pairs
Flower size	large, 2.5–3 cm long	large, ca. 3 × 2 cm	small, 1–1.5 cm long	large, 3 cm deep	large, 3–3.5 cm deep	Small, 2 cm long	large, ca. 3 cm deep, 1.4–2 cm wide	small, 6–8 mm long	small, 1.2–1.4 cm wide	small, 1.2–1.8 cm long
Dorsal petal	oblong-elliptic, ca. 1 cm in diam, as wide as the lateral petals (wing petals), apex obtuse-rounded and mucronulate, both upper and lower bases concave, abaxial midvein carinate.	orbicular, 13–17 mm, concave or 2-cleft at the top, blunt, deeply bifid at the base, with thickened mid-rib on the back, with wings	orbicular, ca. 7 × 7 mm, purple-red spotted, base retuse, apex rostellate, abaxial midvein puberulent	orbicular or obovate, back middle rib keeled, apex mucronulate	reniform, 1.8–2 × ca. 2 cm, apex cornute, abaxial midvein thickened & conspicuously carinate	orbicular, 14 mm, base concave, tip rounded, with thickened mid-rib on the back, broadly winged, wings apically beaked	suborbicular, 1.5–1.8 cm wide, base deeply cordate, apex obtusely cuspidate, abaxial midvein thickened, narrowly carinate, with 2 cristae	orbicular or broadly ovate, 6–7 mm in diam., abaxial midvein thickened, with a small sac or triangular cristate	orbicular, ca. 6 mm, apex mucronulate, abaxial midvein erect, green, carinate, rostellate at middle	orbicular, 6–8 mm diameter, abaxial mid-rib thickened with two erect, stout rostella
Lower sepal	narrowly funnelform, limb 1.5–2 cm long, ca. 1.5 cm deep, mouth obliquely ascending; base gradually narrowed into an incurved long spur, ca. 8 mm wide, apex obtuse and straight	angular, 3–4 cm, mouth is oblique, with a small point, bending inwards or straight from the middle	navicular, ca. 0.8 cm long, mouth vertical, tip acute, abruptly narrowed into an incurved spur	funnel-shaped, ca. 2.5 cm deep, base decurrent into a curved spur of finely spaced	saccate, ca. 3 cm deep, abruptly narrowed into an incurved spur ca. 1 cm; mouth oblique, ca. 1.8 cm wide, tip acute	gable boat-shaped, ascending, 1.5 cm long, mouth obliquely upward, apex acute, base narrowed into a thick, obtuse, slightly recurved spur shorter than the gable	navicular-cornute, 2.5–3 cm deep, mouth oblique, ca. 1.4 cm wide	broadly funnelform, navicular, limb to 9 mm deep, mouth vertical	limb navicular, 1.4–1.5 cm deep, mouth vertical, obtuse	narrowly funnelform, 1.2–1.5 cm long; mouth spreading; base gradually narrowed into an incurved long spur
Spur	curved	curved	incurved, slender	curved	Incurved	slightly curved	erect, stout	curved	involute, slender	curved
Capsule	striate, linear	striate, linear	erect, linear	striate, linear	No data	erect or curved, linear	No data	linear or narrowly clavate	linear or narrowly clavate	striate, linear
Seed	ellipsoid, 3–4 mm long, coats with reticulate ornamentation	oblong, 4 mm long, smooth	ellipsoid, 3.1–3.5 × 1.6–1.9 mm, Some of the seedcoat epidermal cells are elevated significantly higher and arranged in ridges	subglobular, 3 mm in diameter, smooth	No data	brown, ovate-oblong, 3.5–4 mm long, glabrous	black, ovoid-orbicular, ca. 3 × 2 mm	brown, oblong or elliptic, smooth	brown, oblong or elliptic, smooth, shiny	ovoid or ellipsoid, 3.02–3.46 × 1.98–2.51 mm, Seed coat surface reticulate
Inflorescences	2-flowered	2-flowered	2-flowered	1-flowered	2 (or 3)-flowered	2-flowered	3–6-flowered	2-flowered	2- or 3-flowered	2-flowered
Lateral sepals	2, pale green, translucent, lanceolate, glabrous, 0.3–0.6 × 0.1–0.2 cm, apex reflexed	2, green, ovate, 6–8 mm, 3–5 veins, apex acuminate, abaxial midvein thickened	2, ovate, ca. 5 × 3 mm, entire, apex acuminate, abaxial midvein puberulent	2, elliptic, ca. 5 mm, apex rostellate	2, obliquely ovate or suborbicular, 8–9 × 5–6 mm, apex mucronulate, abaxial midvein not thickened	2, ovate, ca. 7 mm, glabrous or puberulent, 3-veined, apex acuminate, mucronulate	2, subulate, ca. 2 mm, 3-veined	2, broadly ovate, 4–5 mm, 3–5-veined, base cordate, apex acute or acuminate	2, ovate, 6–8 mm, herbaceous, apex mucronulate	2, green or purplish-red and spotted with purplish-red, ovate or triangular-ovate, 2.5–3.5 mm long, apex beaked or acute, mid-vein sometimes marginal
Bracts	glabrous	glabrous	puberulent	No data	setose	puberulent	No data	No data	No data	glabrous
Peduncle	4–6 cm	5–10 cm	2–8 cm	4–5 cm	ca. as long as leaves or shorter	2–3 cm	10–20 cm	2–2.5 cm	2–5 cm	4–10 cm

**Figure 1.**
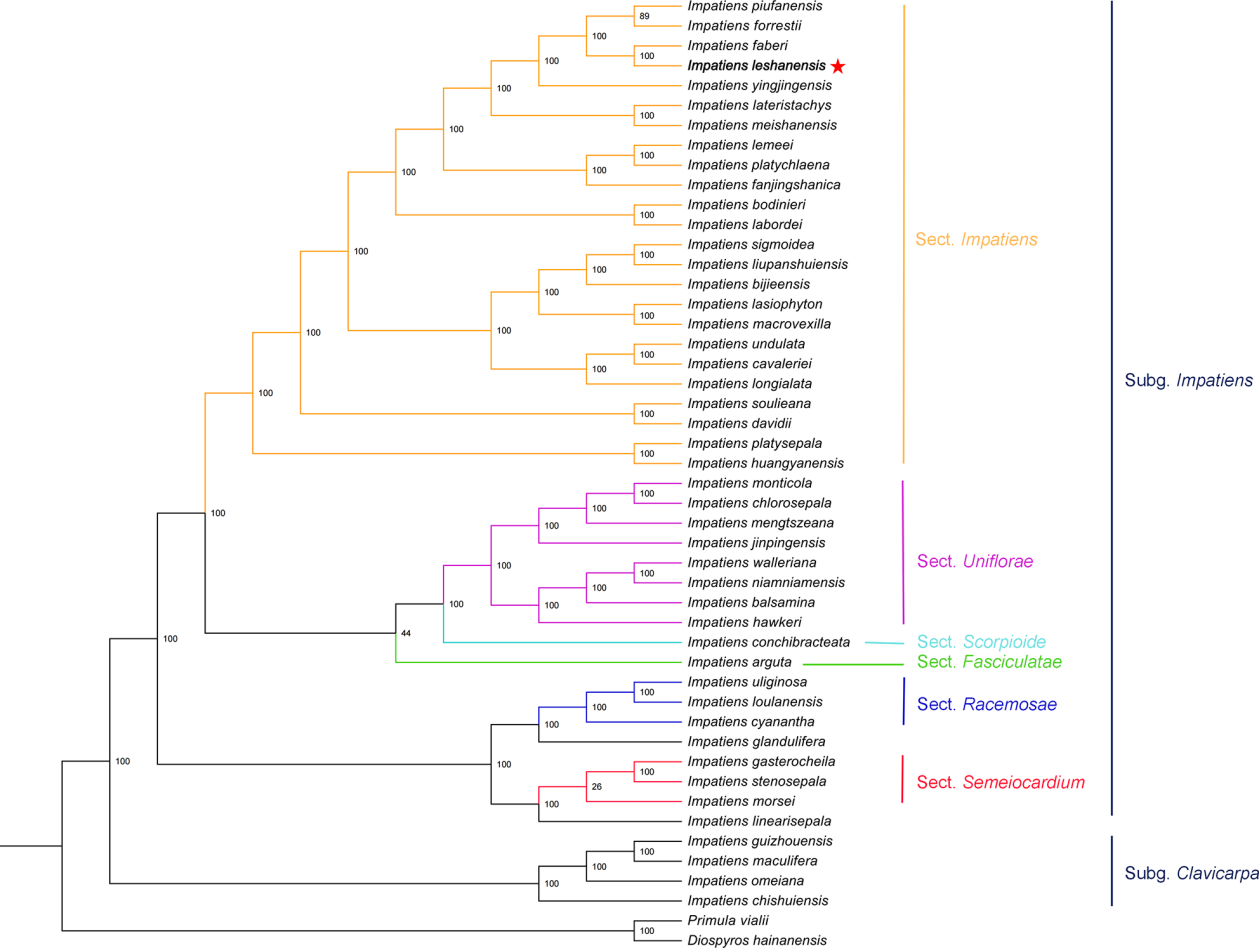
Phylogenetic tree reconstruction of 45 taxa using the ML method, based on complete cp genomes (the phylogenetic position of *Impatiens leshanensis* is marked with a red star).

Maximum Likelihood (ML) phylogenetic trees were inferred using PhyML 3.0 (Guindon et al. 2010), which is accessible via the ATGC bioinformatics platform (https://www.atgc-montpellier.fr/). The analysis was run with the GTRGAMMA model and 1000 bootstrap replicates to assess branch support. Finally, the generated ML phylogenetic trees were edited and visualized using FigTree v1.4.4 (Hu et al. 2018), with the optimized tree presented in Fig. 1.

## Result and discussion

### Taxonomic treatment

#### Impatiens leshanensis

Taxon classification

Plantae

EricalesBalsaminaceae

X.H.Gu, K.Huang & Z.X.Fu sp. nov.

353C3128-11A6-5E51-BAFB-8A6503F081E1

urn:lsid:ipni.org:names:77379454-1

Figs 2, 3, 4

##### Type.

China • Sichuan Province, Leshan City, Jinkouhe District, Gong'an Yi Ethnic Township, Linfeng Village, on moist soil adjacent to mountain roads and streams, elevation 1036.3 m a.s.l., 29.1734, 102.0181, 7 October 2025 (fl.), *Ke Huang, Xingyu Du, Zhixi Fu & Shihao Jiang, Fuzx10888* (**holotype**: SCNU! Barcode 10888a; **Isotype**: SCNU! Barcode 10888b).

**Figure 2.**
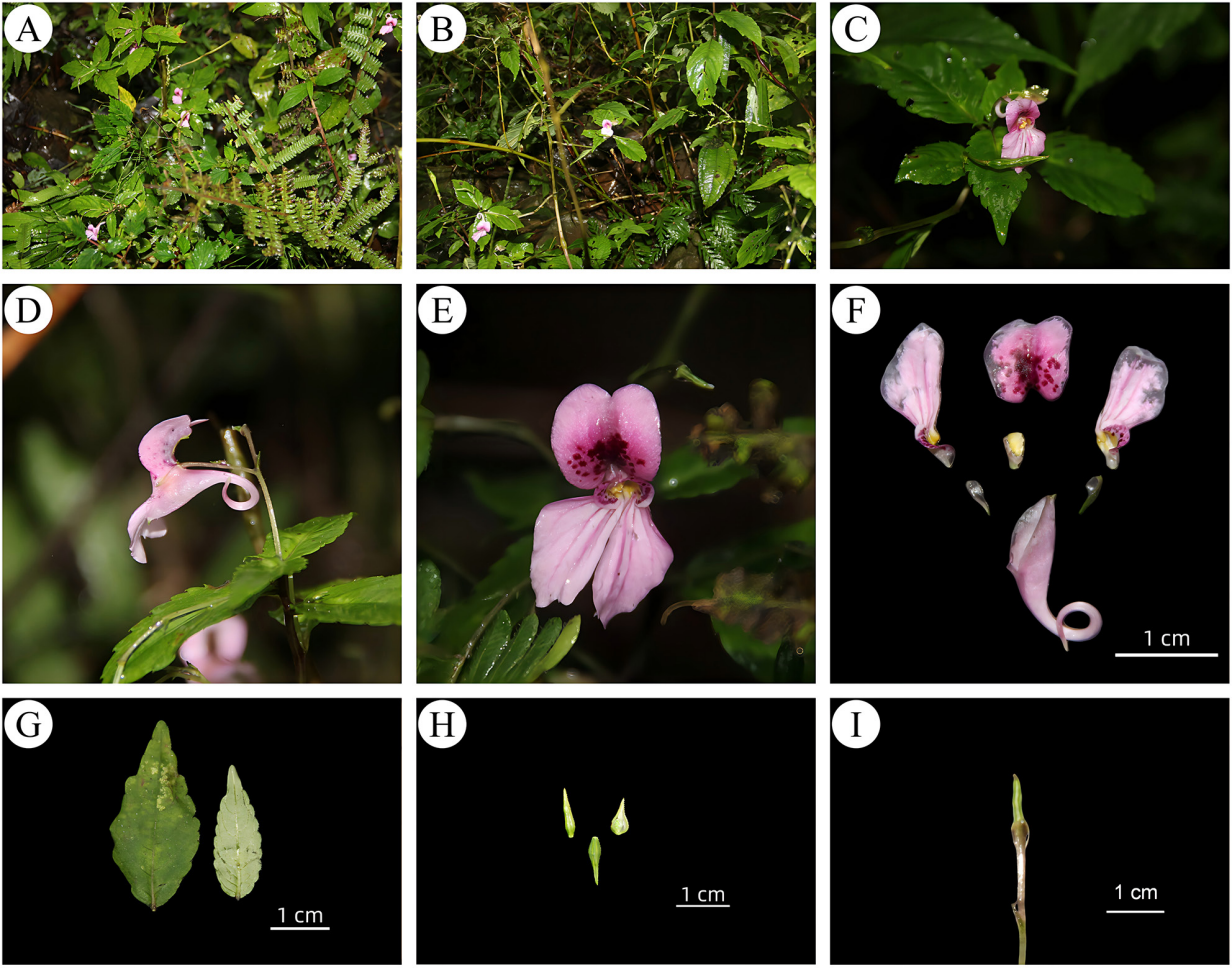
*Impatiens leshanensis* X.H. Gu, K. Huang & Z.X. Fu, sp. nov. A habit B population C plant D flower in lateral view E flower in face view F Flower dissected G leaves H seeds I capsules. Photographs by Xingyu Du.

**Figure 3.**
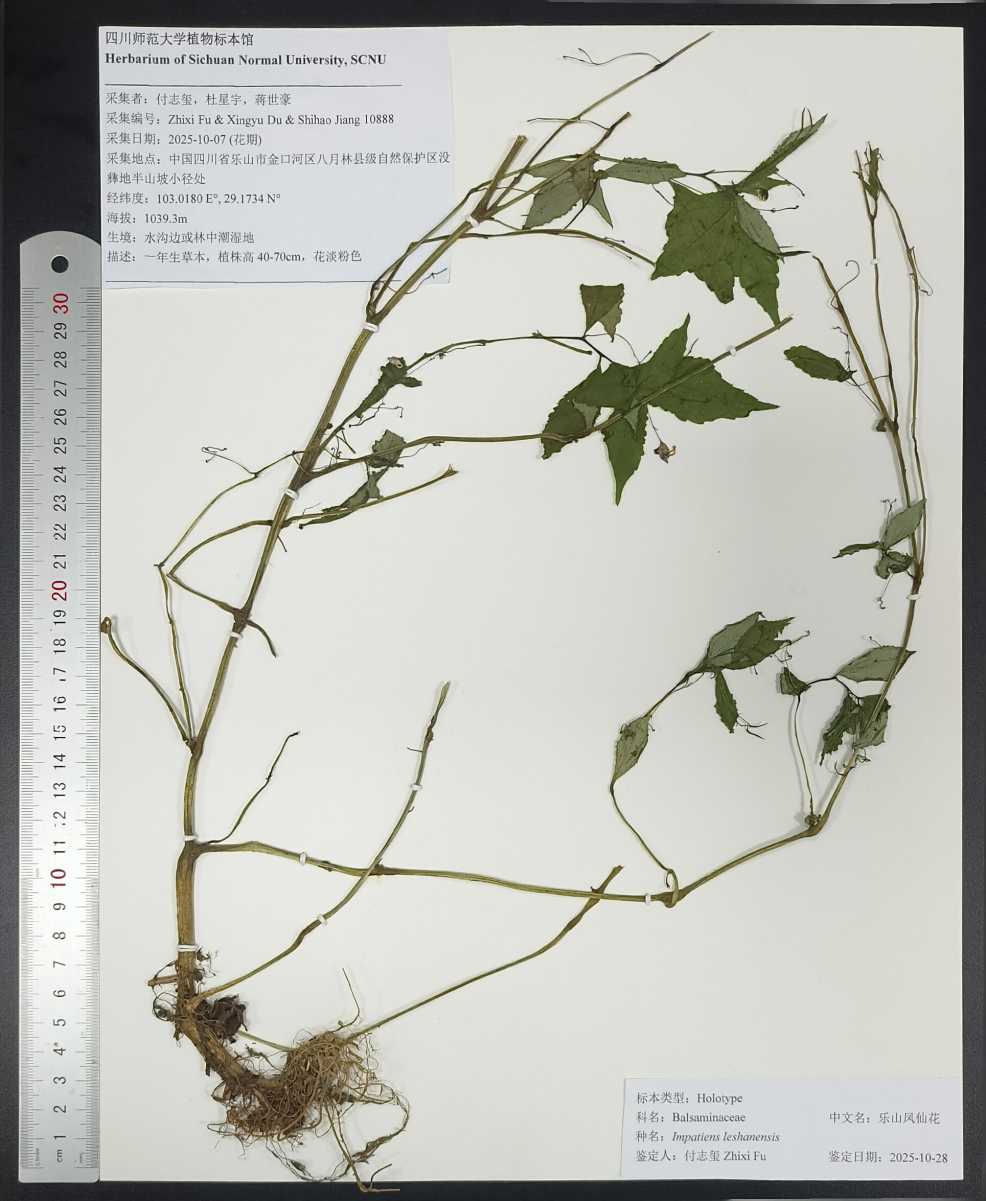
Holotype image of *Impatiens leshanensis* X.H. Gu, K. Huang & Z.X. Fu, sp. nov.

**Figure 4.**
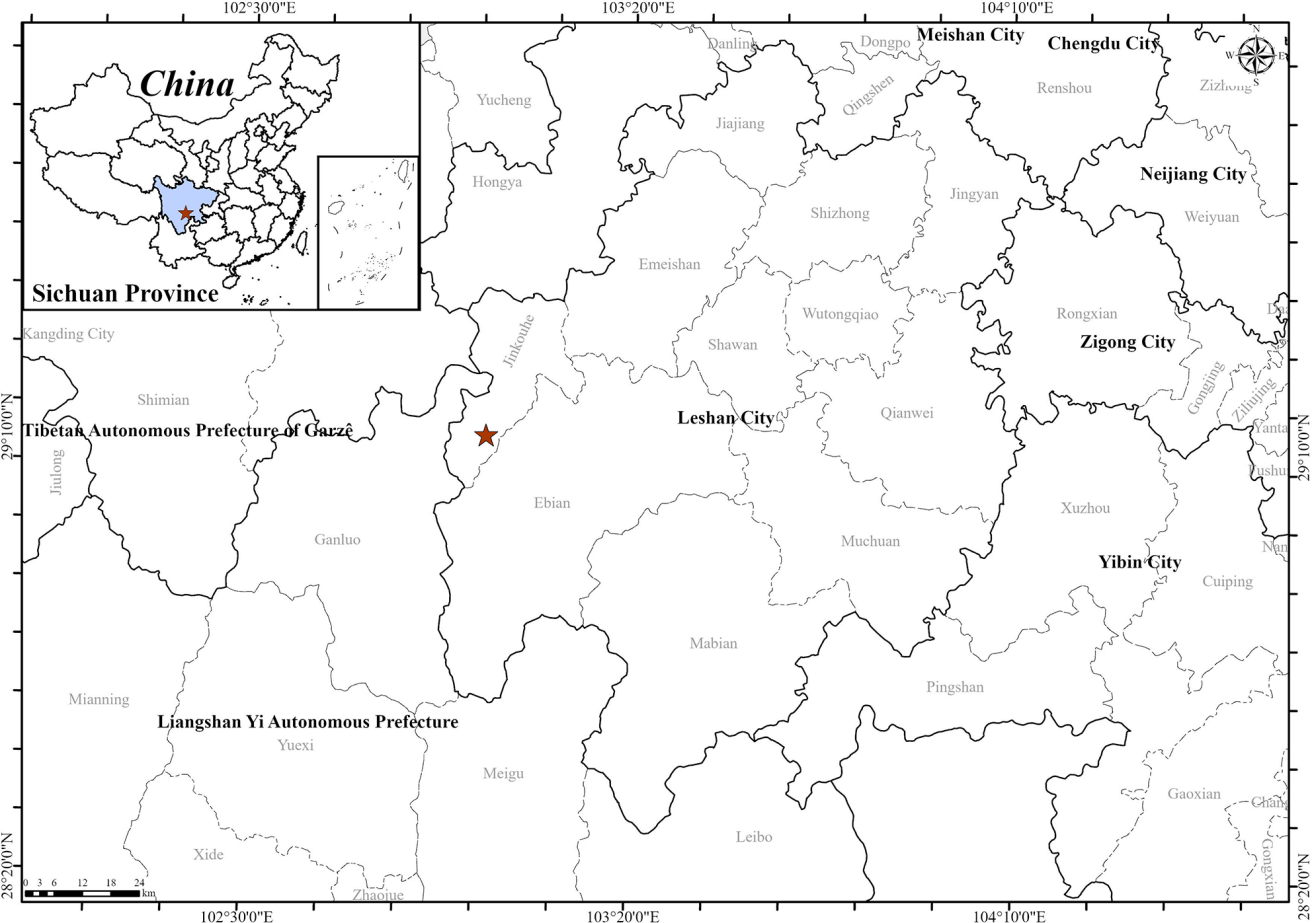
Location of the population of *Impatiens leshanensis* at Jinkouhe District, Leshan City, and Sichuan Province (red star).

##### Diagnosis.

The species of *I. leshanensis* is phylogenetically most closely allied to *I. faberi*, sharing a common ancestral node and morphological synapomorphies, including ovate leaves, two lateral sepals, 2-flowered inflorescences, curved spurs, striate linear capsules, and glabrous bracts. However, it differs significantly in its oblong-elliptic dorsal petal (as wide as the lateral petals/wing petals), narrowly funnelform lower sepal, pale pink flowers with deep red spots and pink stripes, and ellipsoid seeds with reticulate ornamentation (Figs 2, 3).

##### Description.

***Herbs***, annual, 40–70 cm tall, glabrous. ***Stem*** erect, branched at the base. ***Leaves*** alternate, sessile or shortly petiolate. ***Leaf*** blade abaxially green, adaxially grey-green, narrowly ovate or lanceolate, 3–8 × 1–3 cm, glabrous on both surfaces, without setae between teeth, pinnate reticulate veins and ca. 5–7 pairs of conspicuous lateral veins, base cuneate or rounded, margin crenate or crenate-serrate, apex acuminate. ***Inflorescences*** arising from upper leaf axils, 2-flowered. ***Peduncles*** 4–6 cm, slender, erect or slightly curved, glabrous or puberulent, bracteate at the middle or upper-middle part. ***Bracts*** narrowly lanceolate, glabrous. ***Flowers*** pale pink, 2.5–3 cm long, with deep red spots and pink stripes, surface smooth. ***Lateral sepals*** 2, pale green, translucent, lanceolate, glabrous, 0.3–0.6 × 0.1–0.2 cm, apex reflexed. ***Dorsal petal*** oblong-elliptic, ca. 1 cm in diam, as wide as the lateral petals (wing petals), apex obtuse-rounded and mucronulate, both upper and lower bases concave, abaxial midvein carinate. ***United lateral petals *** shortly clawed, 1.2–1.4 cm, 2-lobed. ***Upper petal*** orbicular, apex obtuse-rounded, deep red spotted, ca. 2 mm in diam. ***Lower petal*** dolabriform, auricle linear, elongate, margin undulate, apex obtuse, pink stripes, base pale yellowish-white and translucent. ***Lower sepal*** narrowly funnelform, limb 1.5–2 cm long, ca. 1.5 cm deep, mouth obliquely ascending; base gradually narrowed into an incurved long spur, ca. 8 mm wide, apex obtuse and straight. ***Stamens*** elliptic. ***Ovary*** fusiform, erect. ***Capsules*** striate, linear, 8–10 mm long. ***Seeds*** ellipsoid, 3–4 mm long, with reticulate ornamentation. (Fig. 2)

##### Phenology.

This species of *I. leshanensis* flowers from August to October.

##### Distribution and Ecology.

This new species grows on trails at the mid-slope of Bayuelin city nature reserve, Linfeng Village, Gong'an Yi Ethnic Township, Jinkouhe District, Leshan City, Sichuan Province, China. It may also occur in the nearby area, and is highly likely to inhabit moist soils along streams in forests.

##### Etymology.

The specific epithet "leshanensis" is derived from Leshan City, Sichuan Province, China, referring to the type locality of this new species.

##### Conservation status.

*I. leshanensis* is endemic to the Leshan area of Sichuan Province, China, and is presently known to occur solely in Jinkouhe District with a small population size. Field surveys are insufficient to fully clarify its exact distribution range, population size, and potential threats. Accordingly, we tentatively assess its conservation status as Data Deficient (DD) following the IUCN Red List criteria (IUCN 2024). Its conservation status will be re-evaluated when additional field data and distribution records become available.

##### Notes.

Through critical examination of collected specimens, comparison with type materials of congeneric allied taxa, molecular phylogenetic analysis based on the complete chloroplast genome, and consultation of relevant taxonomic literature, we confirmed the taxonomic position of *I. leshanensis*. Yu et al. (2016) proposed a new taxonomic treatment for *Impatiens* based on morphological and molecular evidence, which includes two subgenera (*Clavicarpa* and *Impatiens*). The subg. *Impatiens* was further divided into seven sections (*Fasciculatae*, *Impatiens*, *Racemosae*, *Scorpioidae*, *Semeiocardium*, *Tuberosae*, and *Uniflorae*). The sect. *Impatiens* is characterized by two-flowered inflorescences, linear capsules, ellipsoid seeds, and seed coats with reticulate ornamentation (Yu et al. 2016). This new species is classified into the sect. *Impatiens* of the subg. *Impatiens*. Morphologically, *I. leshanensis* exhibits several key similarities to *I. baishaensis* (Ding et al. 2017), including annual herbaceous habit, erect and branched stems, alternate leaves, axillary 2-flowered inflorescences, pinkish flowers, linear capsules, and ellipsoid seeds; furthermore, their dorsal petals are analogous in shape and adorned with red spots, the united lateral petals share a comparable morphological pattern, and the upper petals of both species are marked with red spots and pink stripes. However, *I. leshanensis* is readily distinguishable from *I. baishaensis* by the following diagnostic characters: entire plant glabrous (vs. adaxially puberulent leaves, and puberulent abaxial midveins of lateral sepals and dorsal petal); leaf base cuneate or rounded, devoid of stipitate glands (vs. cuneate base with 2–5 stipitate glands); pale green, translucent, lanceolate lateral sepals (0.3–0.6 × 0.1–0.2 cm) with apically reflexed tips (vs. ovate lateral sepals ca. 5 × 3 mm with acuminate apex); and narrowly funnelform lower sepal (1.5–2 cm long) with a retrorse, obtuse-straight spur (vs. navicular lower sepal ca. 0.8 cm long with a slender incurved spur).

This new species, *I. leshanensis*, is similar to *I. faberi*, *I. baishaensis*, *I. piufanensis*, *I. forrestii*, *I. distracta*, *I. lateristachys*, *I. rostellata*, *I. rectirostrata*, and *I. longcanggouensis* (Chen 2001; Chen et al. 2007; Ding et al. 2017; Luo et al. 2026). It differs from *I. faberi* in pale pink flowers with deep red spots and reticulate seeds (vs. purple-red flowers and smooth seeds); from *I. baishaensis* in glabrous whole plant (detailed comparison see above); from *I. piufanensis* in 2-flowered inflorescences and taller height (40–70 cm vs. 20–40 cm); from *I. forrestii* in smaller leaves (3–8 × 1–3 cm vs. 10–15 × 3–5 cm); from *I. distracta* in larger flowers (2.5–3 cm vs. ca. 2 cm) and longer peduncles (4–6 cm vs. 2–3 cm); from *I. lateristachys* in 2-flowered inflorescences (vs. 3–6-flowered raceme); from *I. rostellata* in spotted pale pink flowers (vs. white/pink/yellow/blue flowers); from *I. rectirostrata* in smaller leaves (3–8 × 1–3 cm vs. 10–14 × ca. 5 cm); and from *I. longcanggouensis* in green abaxial leaf surface (vs. purplish-red) and pale pink flowers (vs. white/faintly pinkish). To better distinguish the new species, more details are listed in Table 1.

### Molecular phylogeny

**Figure 5.**
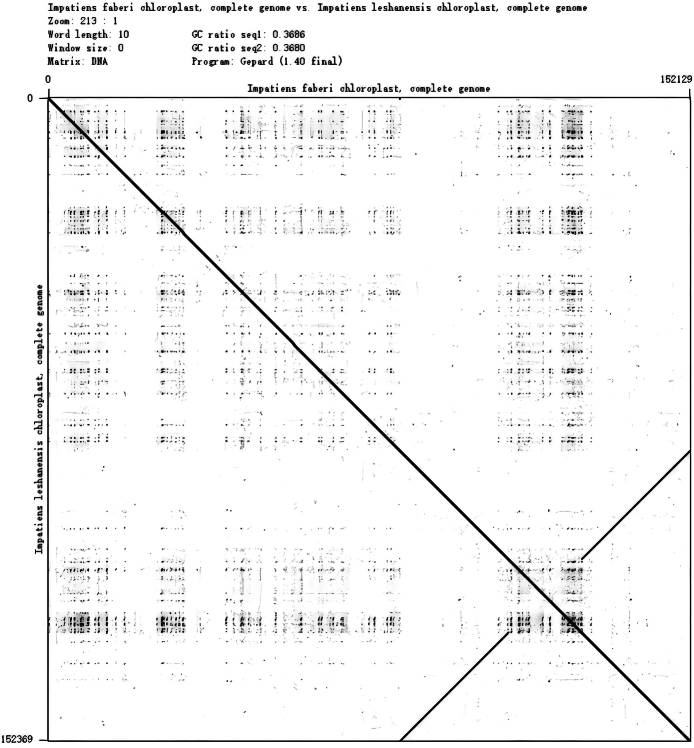
The covariance analyses of two species.

**Figure 6.**
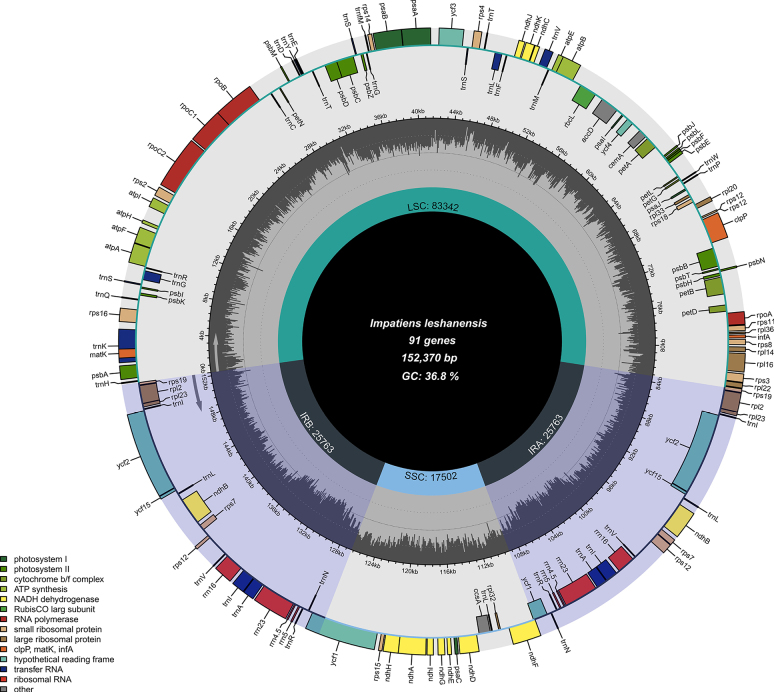
Circular map of *Impatiens leshanensis*. The map of the complete cp genome was generated using Chloroplot.

**Figure 7.**
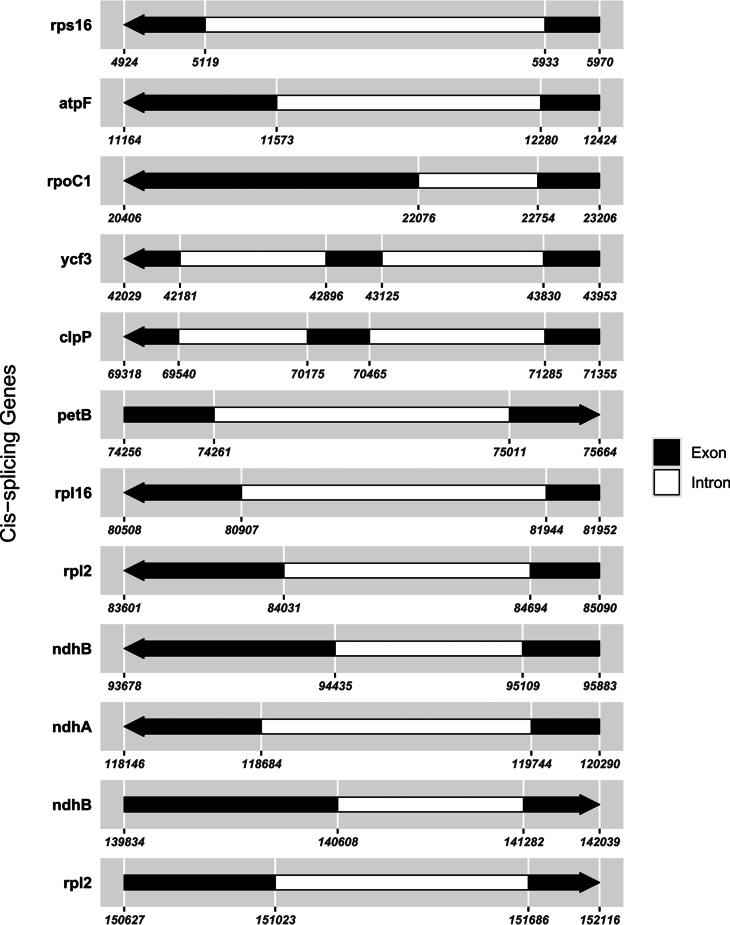
Schematic map of the cis-splicing genes in the cp genome of *Impatiens leshanensis*.

**Figure 8.**
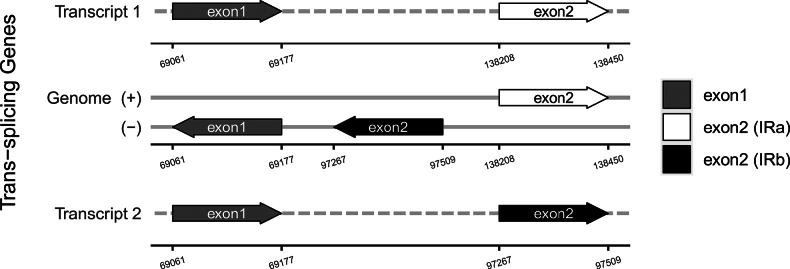
Schematic map of the trans-splicing genes in the cp genome of *Impatiens leshanensis*.

**Figure 9.**
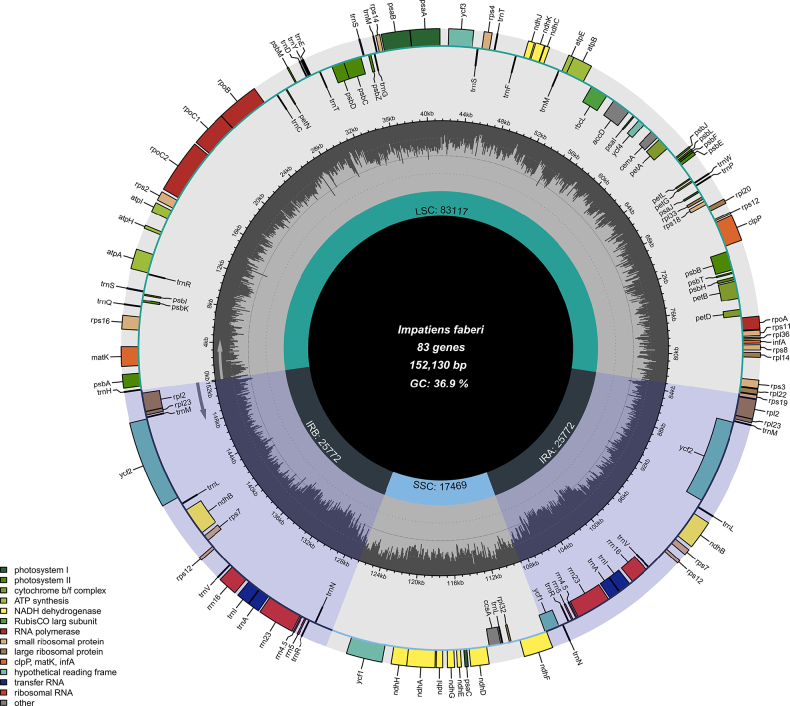
Circular map of *Impatiens faberi* (GenBank accession numbers: PQ652174.1. The map of the complete cp genome was generated using Chloroplot.

**Figure 10.**
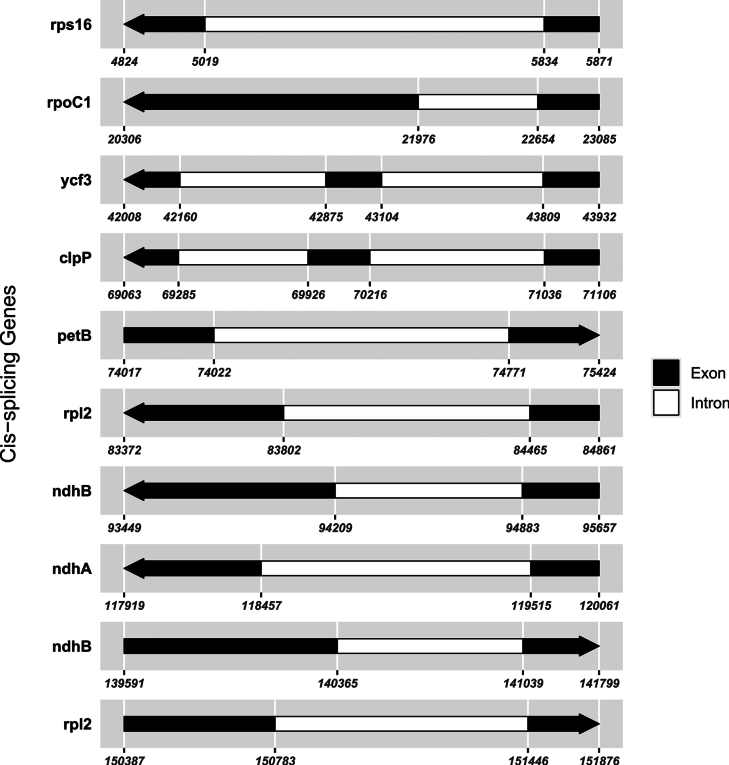
Schematic map of the cis-splicing genes in the cp genome of *Impatiens faberi* (GenBank accession numbers: PQ652174.1).

**Figure 11.**
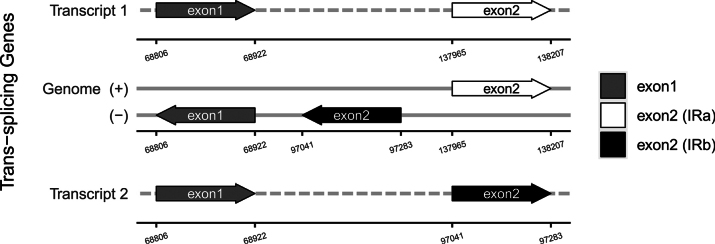
Schematic map of the trans-splicing genes in the cp genome of *Impatiens faberi* (GenBank accession numbers: PQ652174.1).

Phylogenetic analysis was performed by constructing a Maximum Likelihood (ML) tree based on the chloroplast (cp) genome sequences of 45 *Impatiens* species, two outgroups, and the new species (Table 2, Fig. 1), following the classification system of Yu et al. (2016). Phylogenetically, *I. leshanensis* is most closely related to *I. faberi*, with both clustering into a monophyletic clade; *I. piufanensis* and *I. forrestii* form the next closest lineage, constituting a distinct evolutionary branch. Comparative cp genome analysis was conducted between the new species and *I. faberi*, revealing differences in their genomic characteristics (Table 3). The complete cp genome of *I. leshanensis* is 152,370 bp in length with a GC content of 36.8% (Fig. 6), consisting of a large single-copy (LSC) region of 83,342 bp, a small single-copy (SSC) region of 17,502 bp, and inverted repeat (IR) regions totaling 51,526 bp. Its nucleotide composition comprises 47,572 A, 48,719 T, 28,532 C, and 27,547 G residues. In contrast, the complete cp genome of *I. faberi* is 152,130 bp long with a GC content of 36.9% (Fig. 9), including an LSC region of 83,168 bp, an SSC region of 17,589 bp, and IR regions of 51,516 bp, with nucleotide counts of 47,472 A, 48,580 T, 28,500 C, and 27,578 G. Variations in LSC/SSC/IR lengths and nucleotide composition may reflect adaptive evolutionary adjustments between the two species. Genome alignment using Gepard v.2.1.0 (Krumsiek et al. 2007) (Fig. 5) confirmed high synteny between the cp genomes of *I. leshanensis* and *I. faberi*, further supporting their close phylogenetic affinity. Although the core set of cis-splicing chloroplast genes is conserved between the two species, species-specific positional shifts were observed in these genes (Figs 7, 10). A key structural divergence is the presence of cis-splicing genes *atpF* and *rpl16* in *I. leshanensis*, which are absent from *I. faberi*; the *rps12* gene is a trans-splicing gene in both taxa (Figs 8, 11). These genomic variations indicate subtle evolutionary differentiation between these closely related species.

**Table 2.** Accessions sampled for molecular analysis of *Impatiens* and its close relatives.

Species	Family	Genus	GenBank number
*Diospyros hainanensis* Merr.	Ebenaceae	* Diospyros *	NC042160.1
*Primula vialii* Delavay ex Franch.	Primulaceae	* Primula *	NC065387.1
*Impatiens bodinieri* Hook. f.	Balsaminaceae	* Impatiens *	PQ452959.1
*Impatiens chishuiensis* Y.X. Xiong	Balsaminaceae	* Impatiens *	PP724655.1
*Impatiens faberi* Hook. f.	Balsaminaceae	* Impatiens *	PQ652174.1
*Impatiens balsamina* L.	Balsaminaceae	* Impatiens *	NC059942.1
*Impatiens monticola* Hook. f.	Balsaminaceae	* Impatiens *	NC058205.1
*Impatiens cyanantha* Hook. f.	Balsaminaceae	* Impatiens *	NC058204.1
*Impatiens hawkeri* W. Bull	Balsaminaceae	* Impatiens *	NC048520.1
*Impatiens omeiana* Hook. f.	Balsaminaceae	* Impatiens *	NC072171.1
*Impatiens soulieana* Hook. f.	Balsaminaceae	* Impatiens *	PQ452968.1
*Impatiens longialata* E. Pritz.	Balsaminaceae	* Impatiens *	PQ452967.1
*Impatiens sigmoidea* Hook. f.	Balsaminaceae	* Impatiens *	PQ156321.1
*Impatiens cavaleriei* X.X. Bai & R.X. Huang	Balsaminaceae	* Impatiens *	PQ156320.1
*Impatiens lasiophyton* Hook. f.	Balsaminaceae	* Impatiens *	PQ156319.1
*Impatiens liupanshuiensis* X.X. Bai & T.H. Yuan	Balsaminaceae	* Impatiens *	PQ156318.1
*Impatiens labordei* Hook. f.	Balsaminaceae	* Impatiens *	PQ156317.1
*Impatiens bijieensis* X.X. Bai & L.Y. Ren	Balsaminaceae	* Impatiens *	PQ156316.1
*Impatiens huangyanensis* X.F. Jin & B.Y. Ding	Balsaminaceae	* Impatiens *	OR139616.1
*Impatiens walleriana* Hook. f.	Balsaminaceae	* Impatiens *	NC059949.1
*Impatiens stenosepala* E. Pritz.	Balsaminaceae	* Impatiens *	NC059948.1
*Impatiens loulanensis* Hook. f.	Balsaminaceae	* Impatiens *	NC059947.1
*Impatiens linearisepala* S. Akiyama, H. Ohba & S.K. Wu	Balsaminaceae	* Impatiens *	NC059946.1
*Impatiens guizhouensis* Y.L. Chen	Balsaminaceae	* Impatiens *	NC059945.1
*Impatiens fanjingshanica* Y.L. Chen	Balsaminaceae	* Impatiens *	NC059944.1
*Impatiens chlorosepala* Hand. - Mazz.	Balsaminaceae	* Impatiens *	NC059943.1
*Impatiens davidii* Franch.	Balsaminaceae	* Impatiens *	NC058801.1
*Impatiens glandulifera* Royle	Balsaminaceae	* Impatiens *	NC044718.1
*Impatiens piufanensis* Hook. f.	Balsaminaceae	* Impatiens *	NC037401.1
*Impatiens morsei* Hook. f.	Balsaminaceae	* Impatiens *	NC071773.1
*Impatiens conchibracteata* Y.L. Chen & Y.Q. Lu	Balsaminaceae	* Impatiens *	NC071771.1
*Impatiens arguta* Hook. f. & Thomson	Balsaminaceae	* Impatiens *	NC071770.1
*Impatiens platysepala* Y.L. Chen	Balsaminaceae	* Impatiens *	NC068751.1
*Impatiens macrovexilla* var. yaoshanensis S.X. Yu, Y.L. Chen & H.N. Qin	Balsaminaceae	* Impatiens *	NC060668.1
*Impatiens mengtszeana* Hook. f.	Balsaminaceae	* Impatiens *	NC058215.1
*Impatiens uliginosa* Franch.	Balsaminaceae	* Impatiens *	NC059760.1
*Impatiens gasterocheila* Hook. f.	Balsaminaceae	* Impatiens *	PQ452965.1
*Impatiens jinpingensis* Y.M. Shui & G.F. Li	Balsaminaceae	* Impatiens *	PQ452961.1
*Impatiens lemeei* H. Lév.	Balsaminaceae	* Impatiens *	PQ452964.1
*Impatiens maculifera* S.X. Yu & Chang Y. Xia	Balsaminaceae	* Impatiens *	PQ452960.1
*Impatiens niamniamensis* Gilg	Balsaminaceae	* Impatiens *	PQ452962.1
*Impatiens undulata* Y.L. Chen & Y.Q. Lu	Balsaminaceae	* Impatiens *	PQ452969.1
*Impatiens meishanensis* K. Huang & Z.X. Fu.	Balsaminaceae	* Impatiens *	PQ740019.1
*Impatiens yingjingensis* Xin Q. Song, B.N. Song & Biao Yang	Balsaminaceae	* Impatiens *	OR978441.1
*Impatiens forrestii* Hook. f. ex W.W. Sm.	Balsaminaceae	* Impatiens *	PQ877659.1
*Impatiens lateristachys* Y.L. Chen & Y.Q. Lu	Balsaminaceae	* Impatiens *	PQ612862.1
*Impatiens platychlaena* Hook. f.	Balsaminaceae	* Impatiens *	PQ611766.1
***Impatiens leshanensis* X.H. Gu, K. Huang & Z.X. Fu, sp. nov**.	** Balsaminaceae **	*** Impatiens ***	**PX764269.1**

**Table 3.** Comparative analyses of cp genomes between *Impatiens leshanensis* and *I. faberi*.

Species	Genome Size (bp)	LSC (bp)	IR (bp)	SSC (bp)	A	T	C	G	GC Content (%)
*I. leshanensis*	152,37	83,342	51,526	17,502	47,572	48,719	28,532	27,547	36.8
*I. faberi*	152,13	83,168	51,516	17,589	47,472	48,58	28,5	27,578	36.9

The ML phylogenetic tree (Fig. 1) strongly supports the distinct taxonomic status of *I. leshanensis* as a new species. It forms an independent branch that clusters closely with *I. faberi*. Despite their close genetic relationship, multiple lines of evidence confirm their divergence. Although they share certain floral traits, *I. leshanensis* differs from *I. faberi* in key morphological characteristics, including leaf shape, lower sepal structure, and seed ornamentation (Table 1). These morphological disparities are consistent with the observed molecular divergence, suggesting that ecological adaptation or reproductive isolation may have driven the speciation process.

## Supplementary Material

XML Treatment for Impatiens leshanensis

## Additional information

### Artificial Intelligence (AI) use

The authors accept full responsibility for the content of the manuscript, including the disclosure of any use of AI.

No AI tools were used in the preparation of this manuscript.

### Author ORCIDs

X. Du https://orcid.org/0009-0005-2416-3837

Y. Zhang https://orcid.org/0009-0009-0450-2001

S. Jiang https://orcid.org/0009-0009-4790-2160

Q. Wang https://orcid.org/0009-0005-6173-6446

J. Wu https://orcid.org/0009-0004-8515-1437

B. Li https://orcid.org/0000-0002-5131-8639

Y. Chen https://orcid.org/0009-0007-2777-3520

D. Hua https://orcid.org/0000-0002-7778-2608

B. Zhang https://orcid.org/0009-0004-4726-0640

X. Gu https://orcid.org/0009-0004-5175-4379

K. Huang https://orcid.org/0009-0006-2057-5452

Z. Fu https://orcid.org/0000-0002-2789-6287

### Data availability

All of the data that support the findings of this study are available in the main text.
